# Digital phenotyping and digital monitoring technologies for relapse detection in mental health: a systematic review

**DOI:** 10.1186/s12888-026-08033-w

**Published:** 2026-04-07

**Authors:** William Dormechele, Isaac Yeboah Addo, Caleb Boadi, Emmanuel Osei Bonsu, Mercy Oseiwah Adams

**Affiliations:** 1 Navrongo Health Research Centre, Navrongo, Upper East Region Ghana; 2 Centre for Social Research in Health, University of New South Wales, Sydney, Australia; 3 Department of Operations and Management Information Systems, University of Ghana, Accra, Ghana; 4 Department of Epidemiology and Biostatistics, Kwame Nkrumah University of Science and Technology, Kumasi, Ghana; 5 Asesewa Government Hospital, Ghana Health Service, Asesewa, Ghana

**Keywords:** Digital phenotyping, Mental health relapse, Mobile health, Wearable devices, Digital biomarkers, Relapse prediction, Systematic review

## Abstract

**Background:**

Mental health relapse remains a major challenge in the long-term management of psychiatric disorders. Conventional monitoring approaches rely primarily on periodic clinical assessments and self-report measures, which may fail to capture early behavioural changes preceding symptom deterioration. Digital phenotyping, which refers to the continuous collection and analysis of behavioural and physiological data from personal digital devices has emerged as a promising approach for monitoring mental health trajectories and identifying early warning signals of relapse. However, the evidence base remains fragmented, with significant variability in methodologies, populations, and outcome measures, limiting clear conclusions.

**Objective:**

This systematic review synthesises the current evidence on digital phenotyping and related digital monitoring approaches used to detect, predict, or prevent relapse in individuals living with mental health conditions.

**Methods:**

The review followed the Preferred Reporting Items for Systematic Reviews and Meta-Analyses (PRISMA 2020) guidelines and was prospectively registered in PROSPERO (CRD42024561513). A comprehensive literature search was conducted on 17 December 2024 across multiple databases, namely PubMed, Scopus, Web of Science, IEEE Xplore, CINAHL, ACM Digital Library, and Google Scholar. Eligible studies should have examined digital phenotyping or digital monitoring technologies in the context of relapse detection, prediction, or prevention in mental health conditions. Both experimental and observational study designs were included, encompassing randomised and cluster trials, along with pilot, feasibility, and observational monitoring studies. Data were extracted on study characteristics, digital technologies, monitoring modalities, and relapse-related outcomes. Risk of bias for randomised controlled trials was assessed using the Cochrane Risk of Bias (RoB) 2 tool.

**Results:**

Twenty-two studies involving approximately 12,000 participants met the inclusion criteria. Most studies were conducted in high-income countries and evaluated a diverse range of digital monitoring technologies, including smartphone applications, wearable sensing devices, SMS-based interventions, and predictive algorithms. Across studies, digital monitoring technologies demonstrated the potential to identify behavioural signals associated with worsening mental health symptoms and early relapse risk, particularly through smartphone-based monitoring and digital therapeutic platforms. However, the evidence base remains heterogeneous, with many studies focused on feasibility or pilot evaluations rather than validated relapse prediction systems. SMS-based interventions and app-based cognitive behavioural therapy tools generally reported positive findings, whereas wearable devices and predictive algorithm approaches showed mixed findings.

**Conclusion:**

Digital phenotyping and related digital monitoring approaches show promise for improving relapse monitoring and early detection in mental health care. However, the current evidence base remains heterogeneous. Future research should prioritise larger longitudinal studies, standardised relapse definitions, and multimodal monitoring approaches integrating behavioural, physiological, and clinical data to improve the reliability and clinical applicability of digital phenotyping technologies.

**PROSPERO registration:**

CRD42024561513.

**Trial registration:**

Not applicable.

**Supplementary Information:**

The online version contains supplementary material available at 10.1186/s12888-026-08033-w.

## Introduction

Mental health relapse remains one of the most persistent challenges in the long-term management of psychiatric disorders. Relapse typically refers to the recurrence or clinically meaningful worsening of symptoms following a period of remission or clinical stabilisation, and it is associated with significant personal, social, and economic consequences [1]. Recurring episodes of illness often lead to increased healthcare utilisation, functional impairment, stigma, and diminished quality of life for individuals living with mental health conditions [2]. Early identification of relapse risk is therefore a critical component of effective mental health care. Traditional monitoring approaches, such as periodic clinical assessments and patient self-reports, provide valuable information but may fail to capture subtle behavioural changes that occur between clinical visits [3].

Advances in digital technology have created new opportunities for continuous and real-time monitoring of behavioural patterns associated with mental health. One emerging approach is digital phenotyping, which refers to the moment-by-moment quantification of individual-level human behaviour and physiology using data collected from personal digital devices, including smartphones, wearable sensors, and other connected technologies. These systems allow the in-situ collection of behavioural and physiological data such as mobility patterns, sleep, physical activity, communication behaviour, and device interaction patterns [4]. By analysing these data streams using computational and statistical techniques, digital phenotyping approaches aim to identify behavioural signatures associated with mental health states and potential symptom deterioration [5]. In this context, digital phenotyping offers the potential to detect early warning signals of relapse before clinically observable symptoms become severe.

The increasing availability of smartphone sensors, wearable devices, and digital platforms has enabled researchers to explore behavioural signals associated with mental health relapse across a range of psychiatric conditions. For example, evidence has demonstrated that changes in smartphone usage patterns, mobility behaviour, and social interaction metrics may precede relapse episodes in individuals with schizophrenia and other severe mental illnesses [1]. Similarly, multimodal monitoring systems integrating passive behavioural sensing with self-reported symptom data have been proposed as tools for identifying early indicators of psychotic relapse [6]. These developments suggest that passive digital monitoring may provide valuable insights into dynamic behavioural patterns that are difficult to capture through traditional clinical assessments.

Yet, the rapidly evolving discussions on digital mental health technologies has also created a conceptual ambiguity regarding the role of digital phenotyping in mental health care. Digital phenotyping systems are often discussed alongside other digital mental health tools, including digital therapeutic interventions (such as app-based cognitive behavioural therapy or SMS-based psychological support) and clinical prediction or decision-support systems that use algorithmic models to guide treatment decisions [7]. While these technologies may overlap in practice, they represent distinct approaches. Digital phenotyping focuses primarily on passive or semi-passive behavioural data collection and monitoring, whereas digital therapeutic interventions aim to deliver treatment, and predictive decision-support systems. Clarifying these conceptual distinctions is important for accurately evaluating the role of digital phenotyping in relapse prediction and mental health monitoring.

Interestingly, digital phenotyping is increasingly positioned within the broader framework of precision psychiatry, which aims to integrate behavioural, physiological, and environmental data to better understand the dynamic trajectories of psychiatric disorders. Continuous digital monitoring may capture subtle behavioural signals that precede symptom deterioration, such as changes in sleep patterns, activity levels, social rhythms, or communication behaviour. When combined with other clinical or biological markers, these digital behavioural signals may contribute to more personalised and proactive approaches to mental health care [2, 4]. Recent theoretical work has further suggested that digital behavioural monitoring could be integrated with systemic physiological processes to improve understanding of psychiatric relapse dynamics and recovery trajectories [8].

In addition to methodological challenges, digital phenotyping raises important ethical, legal, and governance considerations. The continuous collection of behavioural and physiological data from personal devices involves highly sensitive information, including location patterns, communication behaviours, and daily routines. Concerns regarding data privacy, informed consent, and responsible data governance have therefore become central to discussions about the implementation of digital mental health technologies [2, 9]. As digital phenotyping approaches become more integrated into mental health research and clinical care, ensuring transparent governance frameworks and responsible data practices will be essential for maintaining trust among patients and healthcare providers [4, 8, 10].

Although the potential of digital phenotyping for relapse detection has attracted increasing research attention, the current empirical evidence remains fragmented across heterogeneous study designs, monitoring modalities, and outcome definitions, limiting clear conclusions regarding the reliability and clinical applicability of these approaches. Specifically, existing studies vary widely in terms of sample size, mental health conditions examined, types of digital technologies used, and outcome measures assessed [11, 12]. Some studies focus on passive behavioural monitoring and predictive modelling [6, 12], whereas others evaluate digital interventions designed to improve symptom management or treatment engagement [1, 13]. As a result, there remains limited consensus regarding the extent to which digital phenotyping approaches can reliably detect or predict mental health relapse. A comprehensive synthesis of the available evidence is, therefore, needed to clarify how these approaches are currently being applied in relapse monitoring and to identify key methodological challenges.

This systematic review synthesises the current empirical studies on digital phenotyping and related digital monitoring approaches used to detect, predict, or prevent relapse in mental health conditions. Specifically, the review aims to characterise the types of digital technologies used in relapse monitoring, examine the psychiatric conditions and populations studied, and summarise reported outcomes related to relapse detection, prediction, or prevention. In clarifying the current evidence base and identifying methodological gaps, this review aims to inform future practices and research as well as guiding the effective integration of digital phenotyping approaches into mental health care. 

## Methods

### Study design and registration

This systematic review was conducted in accordance with the Preferred Reporting Items for Systematic Reviews and Meta-Analyses (PRISMA 2020) reporting guidelines and was prospectively registered in the PROSPERO database (CRD42024561513). The completed PRISMA checklist is provided in the Supplementary Material. For the purposes of this review, relapse was defined as the recurrence or clinically meaningful worsening of psychiatric symptoms following a period of remission or clinical stabilisation. Across the included studies, relapse was operationalised using a range of indicators, including deterioration on validated symptom scales, clinician-reported relapse events, hospitalisation related to psychiatric deterioration, or patient-reported worsening of symptoms.

### Search strategy

A comprehensive literature search was conducted on 17 December 2024 to identify relevant studies examining digital phenotyping and digital monitoring approaches in relation to mental health relapse. Searches were performed across multiple electronic databases, namely PubMed, Scopus, Web of Science, IEEE Xplore, CINAHL, ACM Digital Library, and Google Scholar. The detailed search strategies for each database are presented in Supplementary Appendix 1.

The search strategy combined controlled vocabulary and free-text terms related to digital monitoring technologies and mental health relapse. Search concepts included terms such as “digital phenotyping”, “digital biomarkers”, “mobile health”, “mHealth”, “smartphone monitoring”, “wearable technology”, and “digital health”, combined with mental health terms including “depression”, “anxiety”, “bipolar disorder”, “schizophrenia”, “psychosis”, and “mental illness”. These were further combined with relapse-related terms such as “relapse”, “predict”, “detect”, “anticipate”, or “forecast”.

Searches were restricted to studies published between January 2014 and December 2024 to capture contemporary developments in digital mental health technologies. Only peer-reviewed articles published in English were included. Reference lists of included articles were also screened to identify additional relevant studies that may not have been captured in the database searches. Search strings were adapted as necessary to reflect the indexing structure and search interfaces of each database. The full electronic search strategies for all databases are provided in Supplementary Appendix 1 to ensure transparency and reproducibility of the search process.

### Eligibility criteria

Studies were considered eligible for inclusion if they examined digital phenotyping or related digital monitoring technologies in relation to mental health relapse. Given that digital phenotyping approaches are often integrated with digital therapeutic platforms or clinical prediction systems, studies incorporating monitoring components within digital interventions were also considered eligible when they included behavioural monitoring or relapse detection elements. Eligible studies included those investigating people living with mental health conditions or individuals at risk of relapse.

Studies were included if they reported outcomes related to relapse detection, relapse prediction, relapse prevention, or behavioural indicators associated with relapse risk. Both experimental and observational empirical study designs were considered eligible, including randomised controlled trials, cluster randomised trials, pilot studies, feasibility studies, and observational monitoring studies.

Studies were excluded if they did not involve digital monitoring technologies, did not report relapse-related outcomes, or focused exclusively on digital therapeutic interventions without a monitoring or relapse detection component. Review articles, editorials, conference abstracts, and non-empirical publications were also excluded.

### Study selection

Records identified through the database searches were exported to a reference management system and subsequently imported into Rayyan [14], a web-based platform designed to facilitate systematic review screening. Rayyan’s automated duplicate detection function was used to identify potential duplicate records based on similarities in titles, author names, and publication metadata. Identified duplicates were manually verified before removal to ensure accuracy. Prior to formal screening, reviewers conducted a pilot screening of a subset of records to ensure consistent interpretation of the eligibility criteria. Potential duplicates were reviewed manually to ensure accurate removal before screening.

Titles and abstracts were screened independently by two reviewers (WD and CB) using the Rayyan interface. Articles considered potentially eligible were retrieved for full-text assessment. Full-text screening was conducted independently by the same reviewers. Discrepancies between reviewers during both screening stages were resolved through discussion and consensus, with arbitration by a third reviewer (IYA) when necessary. Prior to formal screening, a pilot screening exercise was conducted on a subset of records to ensure consistent interpretation of the eligibility criteria among reviewers.

### Classification of digital approaches

To improve conceptual clarity, digital technologies identified in the included studies were categorised according to their primary function within mental health monitoring systems. Studies were grouped into three broad categories: digital phenotyping systems using passive or semi-passive behavioural data collected from smartphones or wearable devices; digital therapeutic interventions delivered through digital platforms such as mobile applications or SMS-based systems; and predictive or triage algorithms designed to estimate relapse risk or support treatment decision-making. This classification was used to facilitate interpretation of the literature, recognising that some studies incorporated overlapping elements of monitoring, intervention delivery, and predictive modelling. This classification framework was developed iteratively during the synthesis process to facilitate comparison across heterogeneous digital monitoring approaches.

### Outcome measures

The primary outcome of interest was the reported ability of digital phenotyping or digital monitoring technologies to detect, predict, or prevent mental health relapse. Across the included studies, relapse-related outcomes were assessed using a range of indicators, including changes in validated psychiatric symptom scales, clinician-reported relapse events, hospitalisation records, and patient-reported symptom worsening. Several studies also reported behavioural or physiological indicators associated with relapse risk, including changes in mobility patterns, sleep behaviour, physical activity, social interaction patterns, or device usage metrics captured through smartphones or wearable sensors.

### Data extraction

Data from included studies were extracted independently by two reviewers using a structured data extraction form developed for this review. Extracted variables included bibliographic information (author, year of publication, country), study design, participant characteristics, sample size, mental health condition studied, type of digital monitoring technology, intervention characteristics, monitoring modality (e.g., smartphone sensing, wearable devices, SMS systems), relapse-related outcomes, and key study findings. Where necessary, supplementary materials or associated study protocols were consulted to obtain missing methodological details. Extracted data were cross-checked by the review team to ensure accuracy and consistency prior to synthesis. Discrepancies were resolved through discussion and consensus among the authors. Extracted data and study classification decisions were recorded in a structured data extraction sheet developed for this review to ensure transparency and reproducibility of the synthesis process.

### Risk of bias assessment

The methodological quality of included randomised controlled trials was assessed using the Cochrane Risk of Bias 2 (RoB 2) tool [15]. This framework evaluates five domains of potential bias: (1) bias arising from the randomisation process, (2) deviations from intended interventions, (3) missing outcome data, (4) measurement of outcomes, and (5) selection of the reported result. Each domain was rated as “low risk”, “some concerns”, or “high risk”. Studies that were not randomised controlled trials (e.g., pilot, feasibility, or observational studies) were not formally evaluated using RoB 2 but were considered narratively when interpreting the overall strength and limitations of the evidence base.

### Data synthesis

Due to substantial heterogeneity across study designs, digital monitoring modalities, relapse definitions, outcome measures, and follow-up durations, a quantitative meta-analysis was not considered appropriate. Pooling effect estimates across studies would have risked generating misleading summary estimates that would not reflect the methodological diversity of the included investigations. Instead, findings were synthesised using a narrative synthesis approach. Studies were grouped according to the primary type of digital monitoring or intervention technology, including smartphone-based applications, wearable sensing devices, SMS-based interventions, and predictive algorithm or triage systems. Descriptive statistics were used to summarise study characteristics, intervention types, and reported relapse-related outcomes across studies.

## Results

### Study selection

**Fig. 1:**
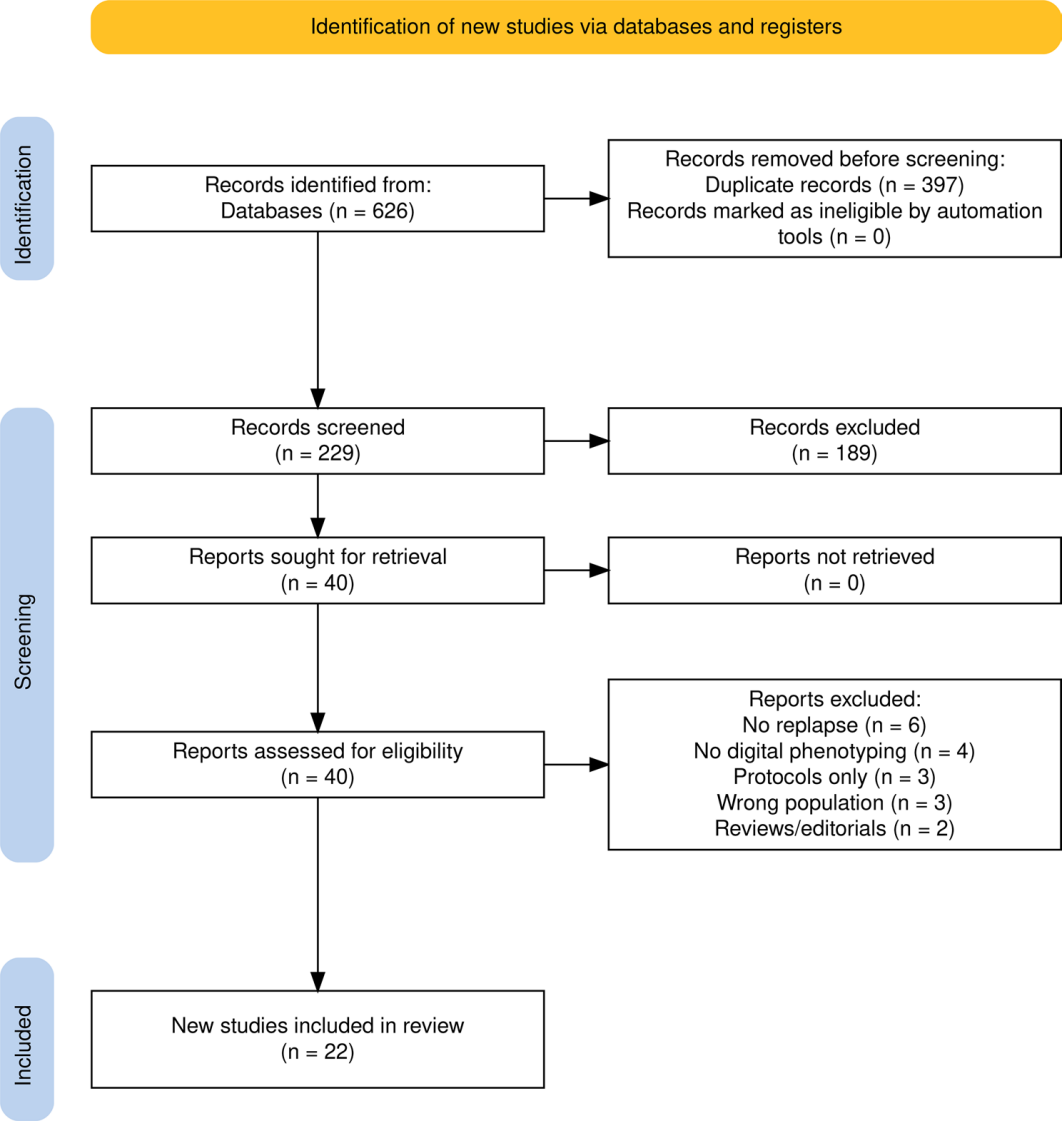
PRISMA flow diagram showing the identification, screening, eligibility assessment, and inclusion of studies in the systematic review

The database search identified 626 records across all databases. After removal of duplicates, 229 unique records remained for title and abstract screening. Of these, 189 records (82.5% of screened records; 189/229) were excluded at the title and abstract stage. The remaining 40 records (40/229, 17.5%) underwent full-text assessment for eligibility. Following full-text review, 18 studies (45.0%; 18/40) were excluded for not meeting the inclusion criteria. Ultimately, 22 studies (55.0%; 22/40) met all eligibility criteria and were included in the final qualitative synthesis. The study selection process is summarised in the PRISMA flow diagram (Fig. 1).

### Study characteristics

The 22 included studies were conducted predominantly in high-income countries (20/22, 90.9%), with the United States accounting for more than half of all studies (12/22, 54.5%). The remaining studies were conducted in Europe, Australia, and India (10/22, 45.5%). Most studies were randomised controlled trials (20/22, 90.9%), while two studies (2/22, 9.1%) used pilot or observational monitoring designs. Sample sizes varied substantially across studies, ranging from 5 participants to 7,884 participants. These studies typically evaluated a diverse group of people with mental health conditions, including individuals with depression, anxiety, schizophrenia, bipolar disorder, eating disorders, and substance use disorders, indicating a broad application of digital phenotyping and related monitoring approaches. Key descriptive characteristics of the included studies are presented in Table 1.

**Table 1:** Descriptive characteristics of studies

Author	Year	Country	Sample size	Aim	Study design
Aguilera et al.	2021	USA	193	To assess the impact of the StayWell SMS program on depression and anxiety symptoms among English and Spanish speakers during COVID-19 social distancing.	Pre-post study
Ali et al.	2023	USA	40	To identify digital biomarkers of motor activity that are implicated in depression, for assessing improvement in depression with antidepressant treatment using wearable device data	Randomized, double-blind, placebo-controlled trial
Anastasiadou et al.	2018	Spain	250	To evaluate the clinical efficacy and cost-effectiveness of integrating a mobile application (TCApp) with standard CBT (Cognitive Behavioural Therapy) compared to standard CBT alone for eating disorder treatment	Multi-centre randomised controlled trial
Andrews et al.	2023	Australia	103	To compare the efficacy of a transdiagnostic biopsychosocial digital mental health program, with or without therapist assistance, for adults with subthreshold symptoms or a diagnosis of anxiety or depression	Adaptive randomized controlled trial
Anmella et al.	2021	Spain	Not reported	To develop a comprehensive machine learning digital support platform (PRESTO) to cost-effectively screen, assess, triage, and provide personalized treatments for anxious and depressive symptoms in primary care	Stepped-wedge cluster randomized controlled trial
Barnett et al.	2018	USA	17	To predict relapse in schizophrenia patients using passively collected smartphone behavioural data to identify warning signs of relapse	Pilot study
Bellón et al.	2023	Spain	720	To design, develop, and evaluate a personalized intervention to prevent depression in primary care using Information and Communication Technologies (ICT), predictive risk algorithms, decision support systems (DSS), and personalized prevention plans (PPPs)	Multicentre cluster randomized controlled trial
Benzo et al.	2022	USA	192	To examine the effects of a tablet-delivered, group-based cognitive-behavioural stress management (CBSM) intervention for reducing symptom burden among men with advanced prostate cancer (APC) and elevated baseline levels of symptom burden	Randomized controlled trial
Ben-Zeev et al.	2017	USA	5	To describe and demonstrate the Crosscheck system, a multimodal data collection system designed to aid in continuous remote monitoring and identification of subjective and objective indicators of psychotic relapse	Description of system and preliminary findings from ongoing randomized controlled trial
Bernstein et al.	2024	USA	77	To explore the use and impact of coaching within a smartphone app-based CBT program for body dysmorphic disorder, focusing on engagement and therapeutic outcomes	Clinical trial
Bhat et al.	2023	India	Not reported	To develop and evaluate a mobile health application, MITHRA, designed to support the identification, initial treatment, and referral of women with depression in community-based organisations (CBOs) in rural India	Pilot cluster randomized controlled trial
Buck et al.	2019	Not reported	61	To evaluate whether smartphone-collected digital measures of social behaviour can provide early indications of relapse events among individuals with schizophrenia	Longitudinal monitoring study
Byrne et al.	2020	Australia	126	To examine whether a mobile health device integrated into a community youth mental health team can improve treatment outcomes for young adults with severe mental illness and predict mental health deterioration	Randomized controlled trial
Fitzsimmons-Craft et al.	2023	USA	90	To evaluate the feasibility and effectiveness of a CBT-based mobile app combined with treatment as usual (TAU), with and without a social networking feature, for women post-acute treatment for anorexia nervosa	Pilot randomized controlled trial
Fitzsimmons-Craft et al.	2021	USA	7884	To test the impact of a mobile mental health platform that uses population-level screening to engage college students in tailored services for anxiety, depression, and eating disorders	Randomized controlled trial
Goulding et al.	2022	USA	205	To evaluate the effectiveness of the LiveWell app in reducing relapse risk, improving quality of life (QOL), and providing self-management strategies for bipolar disorder, while enhancing treatment with real-time assessments and feedback	Single-blind randomized controlled trial (RCT)
Gumley et al.	2020	UK, Australia	120	To establish the feasibility of conducting a definitive cluster randomized controlled trial to compare EMPOWER against treatment as usual, focusing on the prevention of psychosis relapse through early signs monitoring using mobile technology combined with peer support	Feasibility cluster randomized controlled trial
Gumley et al.	2022	UK, Australia	86	To investigate the clinical effectiveness and cost-effectiveness of a digital intervention to recognise and promptly manage early warning signs of relapse in schizophrenia with the aim of preventing relapse	Multicentre, two-arm, parallel-group cluster randomized controlled trial with 12-month follow-up
Gunn et al.	2017	Australia	1320	To test whether using the diamond clinical prediction tool (CPT) to triage and target treatment based on predicted depressive symptom severity is more effective than usual care in reducing depressive symptoms at three months	Stratified individually randomized controlled trial
Lewis et al.	2020	UK	81	To assess the acceptability and impact of a smartphone-based app for real-time symptom monitoring and management in patients with severe mental illness (SMI), focusing on psychosis	Open randomized controlled trial
Redeł et al.	2024	Poland	360	To evaluate the effectiveness of mobile interventions in reducing cravings and preventing lapses in patients with substance use disorder (SUD) using the Nałogometr 20 app	Two-arm participant-blinded randomized controlled trial
White et al.	2021	UK	140	To investigate the effect of insightful notifications, progress visualization, and researcher contact details on both behavioural and experiential engagement with a mobile health data collection platform, RADAR-base	Two-armed randomized controlled trial

### Sample size distribution

Substantial variability was observed in study sample sizes across the 22 included studies. The largest proportion of studies included 50–199 participants (11/22, 50.0%), representing moderate-sized clinical trials typical of early digital mental health interventions.

A further six studies (6/22, 27.3%) included 200–999 participants, while two studies (2/22, 9.1%) involved large samples exceeding 1,000 participants, reflecting large-scale evaluations of digital mental health platforms. In contrast, three studies (3/22, 13.6%) included fewer than 50 participants, representing pilot or feasibility studies designed to test proof-of-concept monitoring systems. The distribution of sample sizes across studies is summarised in Table 2.

**Table 2:** Sample sizes

Sample Size Group	No. of Studies	Percentage	Key References
< 50	3	13.6	Ben-Zeev et al. [6]; Buck et al. [16]; Bernstein et al. [12]
50–199	11	50.0	Fitzsimmons-Craft et al. [17]; Redeł et al. [18]; Ben-Zeev et al. [6]; Gumley et al. [19]; White et al. [20]; Bhat et al. [21]; Byrne et al. [22]; Lewis et al. [23]; Gunn et al. [24]
200–999	6	27.3	Bellón et al. [13]; Anmella et al. [25]; Anastasiadou et al. [26]; Fitzsimmons-Craft et al. [17]; Aguilera et al. [27]; Goulding et al. [28]
≥ 1000	2	9.1	Fitzsimmons-Craft et al. [29]; Gunn et al. [24]; Anmella et al. [25]; Bellón et al. [13]

### Risk of bias assessment

**Fig. 2:**
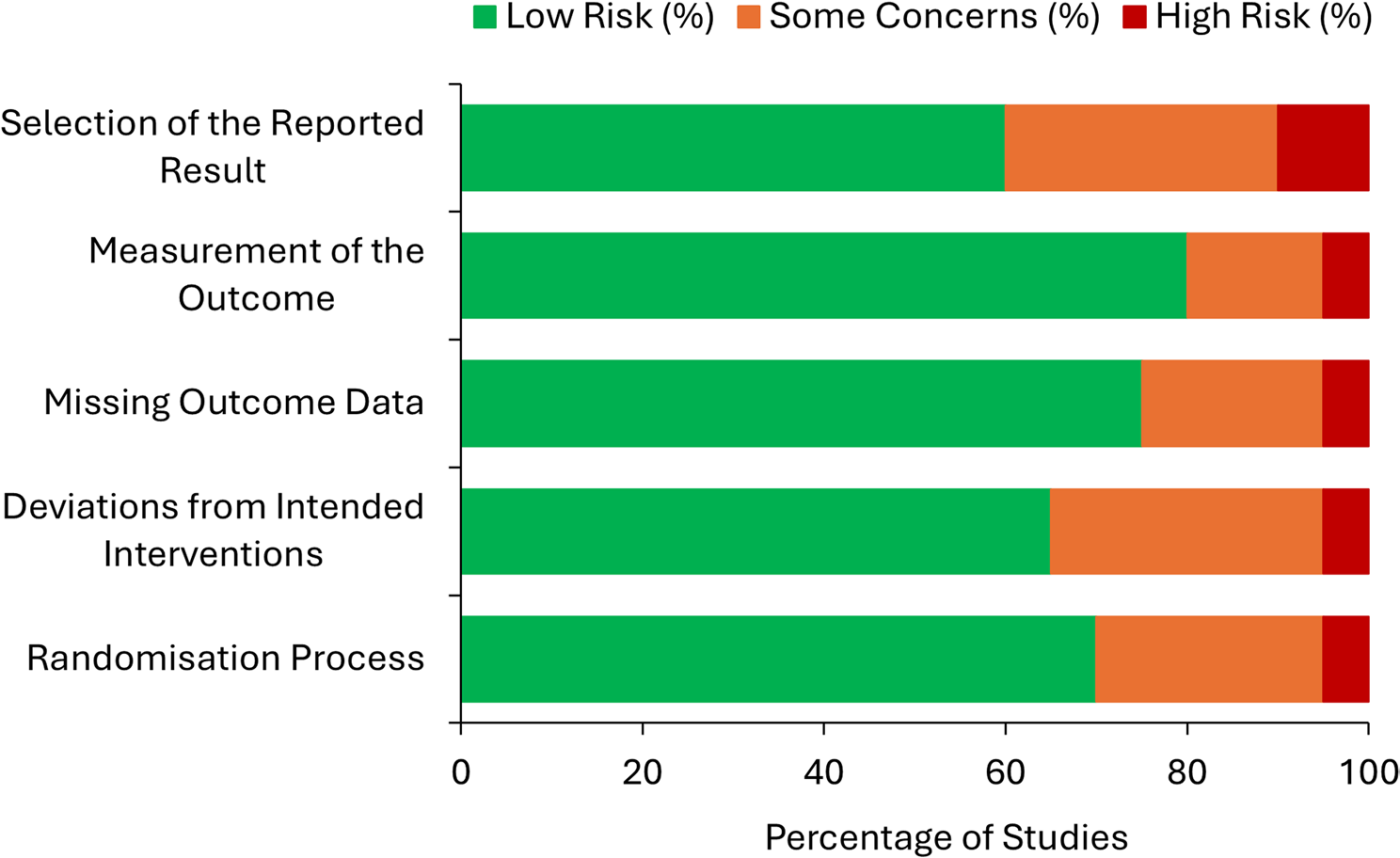
Risk of Bias (RoB 2) assessment across five methodological domains for the 20 randomised controlled trials included in the review

Of the 22 included studies, 20 studies (20/22, 90.9%) were randomised controlled trials and were therefore assessed using the Cochrane Risk of Bias 2 (RoB 2) tool. The remaining two studies (2/22, 9.1%) were pilot or observational studies and were not formally assessed using RoB 2. Across the 20 RCTs assessed, most studies demonstrated low risk of bias across several domains. Specifically, 80% of studies (16/20) were judged to have low risk of bias in the measurement of outcomes domain, while 70% (14/20) were rated as low risk in the randomisation process domain. Higher levels of uncertainty were observed in the domains related to deviations from intended interventions and selection of the reported result, where a greater proportion of studies were rated as having some concerns or high risk of bias. Overall, two studies (2/20, 10.0%) were judged to have high risk of bias in at least one domain. Figure 2 presents a visual summary of domain-level risk assessments across studies.

### Target mental health conditions

The 22 included studies investigated digital phenotyping and digital monitoring technologies across several psychiatric conditions. Depression was the most frequently studied condition (11/22, 50.0%), followed by schizophrenia or psychosis (6/22, 27.3%) and anxiety disorders (5/22, 22.7%). Digital monitoring approaches were also evaluated for eating disorders in three studies (3/22, 13.6%), while bipolar disorder (1/22, 4.5%) and substance use disorder (1/22, 4.5%) were each examined in only one study. Several studies investigated more than one mental health condition; therefore percentages do not sum to 100%. The distribution of mental health conditions across the included studies is summarised in Table 3.

**Table 3:** Target mental health conditions

Mental Disorder Studied	No. of Studies	Percentage (%)	Key References
Depression	11	50.0	Aguilera et al. [27]; Gunn et al. [24]; Anmella et al. [25]; Bellon et al. [13]; White et al. [20]; Bernstein et al. [12]; Lewis et al. [23]; Goulding et al. [19]; Fitzsimmons-Craft [17]; Byrne et al. [22]; Ali et al. [11]
Anxiety	5	22.7	Aguilera et al. [27]; Anmella et al. [25]; Bellon et al. [13]; Fitzsimmons-Craft [17]; Bernstein et al. [12]
Schizophrenia/Psychosis	6	27.3	Ben-Zeev et al. [6]; Buck et al. [16]; Gumley et al. [19]; Gumley et al. [3]; Barnett et al. [1]; White et al. [20]
Eating Disorders	3	13.6	Anastasiadou et al. [26]; Fitzsimmons-Craft et al. [17]; Fitzsimmons-Craft et al. [29]
Bipolar Disorder	1	4.5	Goulding et al. [28]
Substance Use Disorder	1	4.5	Redeł et al. [18]

(*Note: Some studies contributed to multiple mental health conditions and intervention categories; therefore, counts across categories may exceed the total number of included studies.*)

### Effectiveness of digital phenotyping approaches

Among the 22 studies included in this review, 17 studies (17/22, 77.3%) reported positive outcomes, including improved symptom detection, enhanced treatment engagement, or reductions in relapse-related outcomes. Four studies (4/22, 18.2%) reported mixed or neutral findings, while one study (1/22, 4.5%) reported preliminary findings from an early-stage protocol evaluation. However, interpretation of effectiveness should be considered cautiously, as several studies were pilot or feasibility trials and many evaluated usability, engagement, or implementation feasibility rather than validated relapse prediction performance. The distribution of study outcomes is summarised in Table 4.

**Table 4:** Effectiveness of digital phenotyping

Outcome Category	No. of Studies	Percentage (%)	Key References
Positive	17	77.3	Aguilera et al. [27]; Goulding et al. [28]; Anastasiadou et al. [26]; Gunn et al. [24]; Anmella et al. [25]; White et al. [20]; Gumley et al. [19]; Bellón et al. [13]; Fitzsimmons-Craft [17]; Bernstein [12]; Lewis et al. [23]; Bhat et al. [21]; Byrne et al. [22]; Ali et al. [11]; Ben-Zeev et al. [6]; Buck et al. [16]; Redeł et al. [18]
Neutral/Mixed	4	18.2	Byrne et al. [22]; Gumley et al. [3]; Anastasiadou et al. [26]; Ali et al. [11]
Preliminary	1	4.5	Redeł et al. [18] – early protocol stage

### Effectiveness by intervention type

The effectiveness of digital monitoring technologies varied according to the type of intervention employed (Table 5). However, these percentages should be interpreted cautiously because several intervention categories were represented by a small number of studies. SMS-based interventions showed the most consistently positive outcomes (100%), followed by app-based cognitive behavioural therapy platforms (86%). In contrast, predictive algorithms and wearable monitoring systems demonstrated more variable findings, reflecting the early developmental stage of algorithm-driven relapse prediction approaches (Table 5). SMS-based interventions demonstrated the highest effectiveness, with all studies in this category reporting positive outcomes (2/2, 100%). App-based cognitive behavioural therapy (CBT) or self-help tools also demonstrated strong effectiveness, with six of seven studies reporting positive outcomes (6/7, 85.7%). Wearable monitoring technologies showed moderate effectiveness, with two of three studies reporting positive outcomes (2/3, 66.7%). Similarly, predictive algorithms or triage tools demonstrated positive outcomes in three of five studies (3/5, 60.0%), indicating promising but still emerging evidence for algorithm-based relapse prediction approaches. The relationship between intervention type and study outcomes is summarised in Table 5.

**Table 5:** Effectiveness by intervention type

Intervention Type	Total Studies	Positive Outcomes	% Positive	Key References
App-based CBT/Self-Help	7	6	86	Anastasiadou et al. [26]; Goulding et al. [28]; White et al. [20]; Aguilera et al. [27]; Gunn et al. [24]; Fitzsimmons-Craft [17]; Bernstein [12]
Predictive algorithm/triage	5	3	60	Anmella et al. [25]; Bellón et al. [13]; Gunn et al. [24]; Anastasiadou et al. [26]; Ali et al. [11]
SMS-based	2	2	100	Aguilera et al. [27]; Fitzsimmons-Craft [29]
Wearables (actigraphy)	3	2	67	Ben-Zeev et al. [6]; Byrne et al. [22]; Ali et al. [11]

## Discussion

This systematic review synthesised evidence from 22 studies examining digital phenotyping and related digital monitoring approaches for detecting, predicting, or preventing relapse across multiple mental health conditions. Overall, the findings suggest that digital monitoring technologies may offer promising opportunities for identifying behavioural signals associated with mental health deterioration and for supporting early detection of relapse risk. However, the evidence base remains heterogeneous and is characterised by substantial variation in study design, monitoring modalities, outcome definitions, and clinical populations. As a result, while digital phenotyping approaches show promise for enhancing relapse monitoring, their clinical reliability and generalisability remain areas of ongoing investigation.

Across the included studies, digital technologies were used in multiple ways, including passive behavioural sensing from smartphones or wearable devices, digital therapeutic interventions, such as app-based cognitive behavioural therapy (CBT) and SMS-based support programs, and predictive algorithms designed to estimate relapse risk or guide treatment decisions. This diversity reflects the evolving nature of the field, where digital phenotyping technologies are often integrated into intervention delivery platforms or clinical decision-support systems.

Several studies demonstrate the potential of digital behavioural monitoring to detect early warning signs of mental health deterioration. For example, smartphone-based sensing approaches have been used to identify behavioural changes preceding relapse in schizophrenia and other severe mental illnesses [1, 16]. Similarly, multimodal monitoring systems combining self-reported and passive behavioural data have been explored for early relapse detection in psychosis [6]. These studies highlight the potential of passive behavioural signals, including activity patterns, communication behaviour, and mobility data, as indicators of emerging clinical deterioration.

Nevertheless, the evidence base remains highly heterogeneous. Several studies included in this review were pilot trials, feasibility studies, or protocol-driven randomised trials designed primarily to assess usability, engagement, or implementation feasibility rather than validated relapse prediction performance [10]. Although several investigations reported improvements in symptom monitoring or patient engagement, direct comparison across studies remains difficult due to substantial variation in study design, outcome measures, sample sizes, and follow-up durations. As a result, the current evidence should be interpreted as promising but still developing.

Another important consideration is the geographical concentration of the existing evidence base. Most studies included in this review were conducted in high-income countries, particularly the United States and Europe. This geographical concentration raises important questions regarding the generalisability of digital phenotyping approaches to low- and middle-income countries, where mental health service gaps remain substantial but mobile technology adoption is rapidly increasing.

The reviewed literature also exposes the conceptual blurriness between digital phenotyping systems and digital therapeutic interventions. Digital phenotyping primarily involves passive or semi-passive behavioural data collection from personal devices to monitor behavioural patterns associated with mental health states. In contrast, digital therapeutic interventions, such as app-based cognitive behavioural therapy or SMS-based psychological support, aim to deliver treatment or behavioural change strategies. Clarifying this distinction is important because several studies combine monitoring functions with therapeutic components, which may obscure the specific contribution of digital phenotyping to relapse prediction.

From a broader theoretical perspective, digital phenotyping is increasingly situated within the framework of precision psychiatry, which seeks to integrate behavioural, physiological, and environmental data to better understand dynamic mental health trajectories. Continuous behavioural monitoring through smartphones and wearable devices provides a unique opportunity to capture subtle changes in daily functioning that may precede clinical relapse. However, behavioural signals alone may not fully capture the complexity of psychiatric relapse processes. Emerging research suggests that integrating digital behavioural data with physiological or biological indicators may provide a more comprehensive understanding of relapse mechanisms [2, 4, 8].

Despite the promise of digital phenotyping technologies, several methodological challenges remain. First, there is currently no universally accepted definition of relapse across psychiatric disorders, and relapse was operationalised differently across the included studies. Definitions ranged from deterioration on validated symptom scales to clinician-reported relapse events, hospitalisation, or patient-reported symptom worsening. This variability limits direct comparison across studies and highlights the need for more standardised outcome definitions in digital phenotyping research [30].

Second, several studies included relatively small samples or short follow-up periods. Several investigations involved pilot or feasibility designs with fewer than 100 participants, which limits statistical power and the ability to evaluate long-term predictive performance. Larger multi-site longitudinal studies will be necessary to determine whether digital phenotyping systems can reliably predict relapse across diverse populations and clinical contexts.

Third, engagement and adherence remain important challenges. Several studies reported declining adherence to digital monitoring systems over time, particularly for wearable devices or smartphone applications requiring frequent user interaction. Designing digital monitoring systems that minimise user burden while maintaining high-quality data collection will be essential for successful real-world implementation.

Finally, ethical considerations related to privacy, consent, and data governance remain central to the deployment of digital phenotyping technologies. Continuous behavioural monitoring involves the collection of highly sensitive personal data, including location patterns, communication behaviour, and physiological signals. Ensuring robust governance frameworks, transparent consent processes, and responsible data stewardship practices will therefore be critical for the ethical implementation of digital phenotyping technologies [2].

Overall, digital phenotyping and digital monitoring technologies represent a promising direction for improving relapse detection and early intervention in mental health care. However, the current evidence base remains heterogeneous and largely exploratory. Future research should prioritise large-scale longitudinal studies, standardized relapse definitions, and multimodal monitoring approaches that integrate behavioural, physiological, and clinical data to advance the development of reliable relapse prediction systems. 

### Strengths and limitations

A key strength of this review is the comprehensive synthesis of digital phenotyping and related digital monitoring approaches used to detect or prevent relapse across multiple psychiatric conditions. A further strength is the systematic search across multiple interdisciplinary databases, including clinical, technological, and computer science sources, which enabled identification of a diverse body of literature spanning digital mental health interventions, passive behavioural monitoring systems, and predictive algorithmic tools. The review also followed PRISMA 2020 reporting guidelines and used independent screening and data extraction procedures to enhance methodological transparency and reproducibility.

Despite the strengths, several limitations should be considered when interpreting the findings. As noted earlier, the included studies varied substantially in sample size, intervention duration, outcome definitions, and monitoring modalities, which limits direct comparability across studies. Second, several studies were pilot trials, feasibility studies, or protocol-driven investigations, and therefore primarily assessed usability, engagement, or implementation feasibility rather than validated relapse prediction performance. Third, heterogeneity in digital monitoring modalities, including passive smartphone sensing, wearable devices, and digital therapeutic platforms introduces variation in data reliability, user burden, and clinical applicability. Fourth, although most randomised controlled trials demonstrated relatively low risk of bias across several domains, some studies showed concerns related to selective reporting or deviations from intended protocols. Although all 22 studies were included in the results to ensure a comprehensive synthesis of the available evidence, risk of bias assessment was conducted only for the 20 randomised controlled trials using the RoB 2. The remaining two studies employed non-randomised designs and were therefore not amenable to assessment with RoB 2, which is specifically developed for RCTs. Given the absence of a pre-specified alternative tool for non-randomised studies, formal risk of bias assessment was not undertaken for these studies; however, their findings were interpreted with appropriate caution in the narrative synthesis. Finally, publication bias may have favoured studies reporting positive outcomes, potentially inflating overall estimates of effectiveness.

### Implications for research, policy, and practice

The findings of this review highlight several priorities for future research and implementation of digital phenotyping technologies in mental health. From a research perspective, greater methodological harmonisation is needed to improve comparability across studies. Future investigations should adopt standardised definitions of relapse, consistent outcome metrics, and longer follow-up periods to enable more robust evaluation of predictive performance and clinical effectiveness. Large-scale longitudinal studies will also be important for determining whether behavioural signals captured through smartphones or wearable devices can reliably predict relapse across diverse populations and clinical contexts. In addition, the current literature remains heavily concentrated in high-income countries. Expanding research to low- and middle-income countries is critical, particularly given the growing availability of mobile technologies in settings where access to mental health services remains limited.

At the policy level, the expansion of digital phenotyping raises important governance considerations related to privacy, informed consent, and responsible data use. Regulatory frameworks should therefore support the ethical deployment of digital mental health technologies while ensuring appropriate safeguards for sensitive behavioural data collected through personal devices. Clear guidance on data governance, interoperability standards, and clinical evaluation pathways will be necessary to support responsible translation of digital phenotyping systems into routine care.

In clinical practice, digital monitoring tools have the potential to complement traditional mental health services by enabling continuous symptom monitoring and earlier detection of relapse risk. However, successful implementation will require careful integration into existing clinical workflows, adequate clinician training, and patient-centred design approaches that minimise user burden while maintaining data quality. Co-design approaches involving clinicians, patients, and technology developers may be particularly valuable for ensuring that digital phenotyping systems are both clinically meaningful and acceptable to users.

## Conclusion

Digital phenotyping and related digital monitoring technologies show a promising approach for improving the early detection and management of relapse in mental health conditions. Across the included studies, digital monitoring systems demonstrated the potential to identify behavioural signals associated with symptom deterioration and to support relapse prevention strategies. However, the current evidence base remains heterogeneous and is characterised by a substantial proportion of pilot, feasibility, or protocol-driven investigations. As a result, the overall effectiveness of digital phenotyping for reliable relapse prediction remains an emerging area of research rather than an established clinical capability.

Future research should prioritise large-scale longitudinal studies, standardised definitions of relapse, and multimodal monitoring systems that integrate behavioural, physiological, and clinical data. Addressing ethical considerations related to privacy, consent, and data governance will also be essential for ensuring responsible implementation of these technologies. Strengthening the methodological rigour and clinical validation of digital phenotyping approaches will be critical for determining their long-term role in supporting personalised and proactive mental health care.

## Supplementary Information

Below is the link to the electronic supplementary material.

Supplementary Material 1

## Data Availability

All data generated or analysed during this systematic review are derived from previously published studies, which are cited within the manuscript. The full dataset supporting this article is available from the corresponding author upon reasonable request.

## Abbreviations

CBT: Cognitive Behavioural Therapy
mHealth: Mobile Health
RCT: Randomised Controlled Trial
RoB 2: Risk of Bias 2
PRISMA: Preferred Reporting Items for Systematic Reviews and Meta-Analyses
