# Supplementary material for: Digital phenotyping and digital monitoring technologies for relapse detection in mental health: a systematic review
Source: BMC Psychiatry. 2026 Apr 7;26:410. doi: 10.1186/s12888-026-08033-w (PMC13188289; doi:10.1186/s12888-026-08033-w)
Supplement: Supplementary file 1 — Supplementary Material 1 [file 12888_2026_8033_MOESM1_ESM.docx]

The search strategy was applied across multiple major databases, including PubMed, Scopus, Web of Science, and Google Scholar. Below are the various search terms used in the selected databases.

**Scopus**

( TITLE-ABS-KEY ( "digital phenotyping" OR "digital biomarkers" OR "mobile health" OR "mHealth" OR "eHealth" OR "telehealth" OR "smartphone monitoring" OR "wearable technology" OR "digital health" ) AND TITLE-ABS-KEY ( "mental health" OR "mental illness" OR "psychiatric disorder*" OR "depression" OR "anxiety" OR "bipolar disorder" OR "schizophrenia" OR "PTSD" OR "psychosis" OR "mental health relapse*" OR "psychiatric relapse*" ) AND TITLE-ABS-KEY ( "predict*" OR "forecast*" OR "anticipate" OR "detect*" ) AND TITLE-ABS-KEY ( "RCT" OR "randomized controlled trial*" OR "randomised controlled trial*" ) ) AND PUBYEAR > 2013 AND PUBYEAR < 2025

**PubMed**

(("digital phenotyping" OR "digital biomarkers" OR "mobile health" OR "mHealth" OR "eHealth" OR "telehealth" OR "smartphone monitoring" OR "wearable technology" OR "digital health") AND ("mental health" OR "mental illness" OR "psychiatric disorder*" OR "depression" OR "anxiety" OR "bipolar disorder" OR "schizophrenia" OR "PTSD" OR "psychosis" OR "mental health relapse*" OR "psychiatric relapse*") AND ("predict*" OR "forecast*" OR "anticipate" OR "detect*") AND ("RCT" OR "randomized controlled trial*" OR "randomised controlled trial*") AND (("2014/01/01"[PDat] : "2024/12/31"[PDat]))).

**Web of Science**

AB= ("digital phenotyping" OR "digital biomarkers" OR "mobile health" OR "mHealth" OR "eHealth" OR "telehealth" OR "smartphone monitoring" OR "wearable technology" OR "digital health" ) AND AB= ( "mental health" OR "mental illness" OR "psychiatric disorder*" OR "depression" OR "anxiety" OR "bipolar disorder" OR "schizophrenia" OR "PTSD" OR "psychosis" OR "mental health relapse*" OR "psychiatric relapse*" ) AND AB= ( "predict*" OR "forecast*" OR "anticipate" OR "detect*" ) AND AB= ( "RCT" OR "randomized controlled trial*" OR "randomised controlled trial*" ) AND PY=(2014-2024)

**Google Scholar**

( "digital phenotyping" OR "digital biomarkers" OR "mobile health" OR "mHealth" OR "eHealth" OR "telehealth" OR "smartphone monitoring" OR "wearable technology" OR "digital health" ) AND ( "mental health" OR "mental illness" OR "psychiatric disorder*" OR "depression" OR "anxiety" OR "bipolar disorder" OR "schizophrenia" OR "PTSD" OR "psychosis" OR "mental health relapse*" OR "psychiatric relapse*" ) AND ( "predict*" OR "forecast*" OR "anticipate" OR "detect*" ) AND ( "RCT" OR "randomized controlled trial*" OR "randomised controlled trial*" ) )
